# Molecular Genetic Analysis and Evolution of Segment 7 in Rice Black-Streaked Dwarf Virus in China

**DOI:** 10.1371/journal.pone.0131410

**Published:** 2015-06-29

**Authors:** Yu Zhou, Jianfeng Weng, Yanping Chen, Jirong Wu, Qingchang Meng, Xiaohua Han, Zhuanfang Hao, Mingshun Li, Hongjun Yong, Degui Zhang, Shihuang Zhang, Xinhai Li

**Affiliations:** 1 Institute of Crop Science, Chinese Academy of Agricultural Sciences, Zhongguancun South Street, Haidian District, Beijing, China; 2 Institute of Food Crops and Institute of Food Safety and Detection, Jiangsu Academy of Agricultural Sciences, Nanjing, Jiangsu Province, China; 3 Institute of Food Crops, Henan Academy of Agricultural Sciences, Zhengzhou, Henan Province, China; Oklahoma State University, UNITED STATES

## Abstract

Rice black-streaked dwarf virus (RBSDV) causes maize rough dwarf disease or rice black-streaked dwarf disease and can lead to severe yield losses in maize and rice. To analyse RBSDV evolution, codon usage bias and genetic structure were investigated in 111 maize and rice RBSDV isolates from eight geographic locations in 2013 and 2014. The linear dsRNA S7 is A+U rich, with overall codon usage biased toward codons ending with A (A3s, S7-1: 32.64%, S7-2: 29.95%) or U (U3s, S7-1: 44.18%, S7-2: 46.06%). Effective number of codons (Nc) values of 45.63 in S7-1 (the first open reading frame of S7) and 39.96 in S7-2 (the second open reading frame of S7) indicate low degrees of RBSDV-S7 codon usage bias, likely driven by mutational bias regardless of year, host, or geographical origin. Twelve optimal codons were detected in S7. The nucleotide diversity (π) of S7 sequences in 2013 isolates (0.0307) was significantly higher than in 2014 isolates (0.0244, *P* = 0.0226). The nucleotide diversity (π) of S7 sequences in isolates from Jinan (0.0391) was higher than that from the other seven locations (*P* < 0.01). Only one S7 recombinant was detected in Baoding. RBSDV isolates could be phylogenetically classified into two groups according to S7 sequences, and further classified into two subgroups. S7-1 and S7-2 were under negative and purifying selection, with respective *Ka/Ks* ratios of 0.0179 and 0.0537. These RBSDV populations were expanding (P < 0.01) as indicated by negative values for Tajima's D, Fu and Li's D, and Fu and Li's F. Genetic differentiation was detected in six RBSDV subpopulations (P < 0.05). Absolute *Fst* (0.0790) and *Nm* (65.12) between 2013 and 2014, absolute *Fst* (0.1720) and *Nm* (38.49) between maize and rice, and absolute *Fst* values of 0.0085-0.3069 and *Nm* values of 0.56-29.61 among these eight geographic locations revealed frequent gene flow between subpopulations. Gene flow between 2013 and 2014 was the most frequent.

## Introduction

Rice black-streaked dwarf virus (RBSDV), a member of the genus *Fijivirus* in the family *Reoviridae*, causes maize rough dwarf disease (MRDD) and rice black-streaked dwarf disease (RBSDD), which lead to severe yield losses in maize and rice in East Asia [1, 2]. Variability, codon usage and nucleotide composition bias, recombination, selection pressure, and population genetic structure can each affect the evolution of a virus [3–7]. Therefore, we investigated the population codon usage bias and genetic structure of RBSDV in 111 MRDD and RBSDD isolates (S1 Table) sampled from eight geographic locations in 2013 and 2014. These locations were mainly in the Yellow and Huai River summer maize-growing regions of China, where the MRDD prevailed, including Henan, Shandong, Jiangsu, Hebei provinces and Beijing.

RBSDV has icosahedral, double-layered particles with a diameter of 75–80 nm that contain ten linear dsRNAs (S1-S10) that range in size from 1.8 to 4.5 kb [2, 8–11]. The dsRNA S7 is comprised of two ORFs designated S7-1 and S7-2 that encode the proteins P7-1 and P7-2, respectively. P7-1 is a nonstructural protein comprised of 363 amino acids (with a molecular mass of 41.0 kDa) that causes male sterility due to nondehiscent anthers in *Arabidopsis* [12]. P7-2 is a nonstructural protein comprised of 309 amino acids with a molecular mass of 36 kDa that interacts with SKP1, a core subunit of SCF ubiquitin ligase [13]. Although P7-1 and P7-2 exhibit many characteristics consistent with a role in virus replication, the genetic structure and codon usage bias of their encoding dsRNAs have not yet been elucidated. Further, the interactions of host plants with RBSDV should be examined to gain insights into the evolution of the S7 dsRNA.

Studying the nucleotide composition of these viral molecules, and the extent and causes of biases in their codon usage is essential to understanding the evolution of RBSDV, particularly to detect any interplay between the virus and the cells or immune responses of its hosts [4]. Studies have revealed complicated patterns of nucleotide composition and codon usage bias (CUB) in some viruses, but the forces shaping their evolution have not been illuminated [4]. Codon usage bias refers to the phenomenon wherein synonymous codons do not appear with equal frequencies in protein sequences. Synonymous codon usage has been studied in a wide variety of organisms, including prokaryotes, eukaryotes, and viruses [14]. CUB occurs in higher organisms, microorganisms, and in some human and animal viruses [15–18]. Among plant viruses, there have been studies on sobemovirus [19], citrus tristeza virus [20], and soybean dwarf virus [21]. However, there has been little research into CUB in RBSDV or other reoviruses to date [22].

Analyses of the population genetic structure of viruses can provide better understanding of their molecular evolution. Mechanisms that drive the evolution and geographical dispersion of plant viruses have been studied in some viruses [6] including turnip mosaic virus (TuMV) [23], tobacco vein banding mosaic virus (TVBM) [24], rice yellow mottle virus (RYMV) [25], tomato spotted wilt virus (TSWV) [26], soybean mosaic virus (SMV) [27], wheat yellow mosaic virus (MYMV) [28], fig mosaic virus (FMV) [29], cucumber mosaic virus (CMV) [30], and potato virus M (PVM) [31]. Evidence of population genetic structure has previously been reported for the dsRNA sequences S8 [1], S9 [32], and S10 [1, 2] from RBSDV.

However, analyses of the genetic structure and codon usage bias of the RBSDV S7 dsRNA had not previously been performed. In the present study, the genetic structure and codon usage bias of 111 RBSDV S7 sequences from maize and rice hosts from eight geographic locations in 2013 and 2014 were analysed. Our findings provide further insights into the evolution of RBSDV based on molecular genetic analysis of the S7 dsRNA.

## Materials and Methods

### Sampling of virus isolates

Maize and rice plants with symptoms of rough dwarf disease of Beijing (I) were collected from the experimental field of Chinese Academy of Agricultural Sciences. In Tangshan (II), plants were collected together with Wen-Yue Tong of Tangshan Agricultural Reseach Institutes. In Baoding (III), plants were collected together with Dr. Jie Shi and Dr. Bo Li of Hebei Academy of Agriculture and Forestry Sciences. In Jinan (IV), plants were collected together with Dr. Zhao-Dong Meng and Dr. Qi Sun from Shandong Academy of Agricultural Sciences. In Jining (V), plants were collected together with Zhao-Wen Sun of Jining Agricultural Reseach Institutes. In Zhengzhou (VI), plants were collected together with Dr. Shuang-Gui Tie and Dr. Xiao-Hua Han of Henan Academy of Agricultural Sciences. In Yancheng (VII) and Nanjing (VIII), plants were collected together with Dr. Yan-Ping Chen of Jiangsu Academy of Agricultural Sciences. In this study, our maize and rice plants were not cultivated on private land. Our study involved no specific permissions for these locations/activities, because our plant materials were all collected together with the scientific researchers of local institutions in the experimental fields of every academy of agricultural sciences. Our study did not involve endangered or protected species.

A total of 111 maize or rice plants with symptoms of maize rough dwarf disease or rice black-streaked dwarf disease were collected from eight areas in which these diseases prevailed in 2013 and 2014 (S1 Table). Nine plants were collected from Beijing, 21 from Hebei, 33 from Shandong, 25 from Henan, and 23 from Jiangsu. Rice plants were also harvested from near the same locations in which maize was also cultivated in Baoding (III), Jining (V), Zhengzhou (VI), and Nanjing (VIII). These virus-infected plant leaves were frozen in liquid nitrogen and stored at -80 °C. A total of 76 maize isolates from eight geographic locations (from I through VIII), and 35 rice isolates from four geographic locations (II, V, VI, and VIII) (S1 Table) were processed and used for analyses of RBSDV S7 sequences.

### RNA extractions, RT-PCR, and sequencing

RBSDV dsRNA was extracted from individual maize and rice isolates following previously described methods [9, 33, 34]. The quality and integrity of the dsRNA were assessed on 1.2% native agarose gels and the dsRNA concentrations were estimated using a NanoDrop 2000 spectrophotometer (Thermo Scientific, USA). First-strand cDNA was synthesized using a Fast Quant RT Kit (TIANGEN, China), and PCR products were amplified with two pairs of S7-specific primers (S2 Table) using KOD-Plus-Neo enzyme (TOYOBO, Japan). These products were then sequenced at the AuGCT DNA-SYN Biotechnology Company (Beijing, China) using the dideoxy chain-termination method. For partial S7 sequences, three independent PCR reactions were sequenced to confirm sequencing quality. The sequence data was assembled and analyzed using DNAMAN and Jemboss1.5 software (EMBOSS, Cambridge, UK) [35].

### Analysis of codon usage bias in S7-1 and S7-2 sequences

Codon usages in P7-1 and P7-2 were assessed using the program Codon W 1.4.4 (http://sourceforge.net/projects/codonw/). The effective number of codons (*Nc* value) represents the bias towards synonymous codons but does not pertain to amino acid composition or codon number [36, 37]. *Nc* values for different genes or isolates ranged from 20 (when one codon is used per amino acid) to 61 (when all possible codons are used equally). Highly expressed genes tend to have high codon bias with low *Nc* values [38]. GC3_S_ denotes the frequency of G+C, and the expressions A3_S_, U3_S_, G3_S_, or C3_S_ indicate the frequencies of A, U, G, or C, respectively, at synonymous third-base positions.

The codon adaptation index (*CAI*) was used to measure the extent of codon bias in expressed genes [39, 40], S7-1 and S7-2 in the present study. The value of *CAI* ranges from zero to one, where a value of one indicates high codon usage bias and potential expression level [40]. The codon bias index (*CBI*) was used to estimate the proportion of preferred codons [41]. When the *CBI* value is one, only preferred codons are used for all triplets in the mRNA, which would indicate a nonrandom process. In contrast, negative values for *CBI* indicate that nonpreferred codons are used more often than expected.

To determine the preferred codons for the S7-1 and S7-2 sequences, the value for relative synonymous codon usage (*RSCU*) was calculated using 111 sequences from 111 isolates. *RSCU* is the ratio of the observed to the expected codon frequency, assuming that all synonyms for that amino acid have an equal chance of being used. There is positive codon usage bias when the value of *RSCU* is greater than one, and relatively negative codon usage bias when *RSCU* is less than one. When *RSCU* equals one, a codon has been chosen randomly [42].

Five percent of the total genes with the highest and lowest *CAI* values were defined as the high- and low-expression datasets respectively, and were selected to determine optimal codons. Codon usage was compared using a Chi-squared contingency test of groups, defining codons whose frequency of usage was significantly higher (*P* < 0.01) in the high-expression dataset than in the low-expression dataset as the optimal codons [43].

### Sequence variants and nucleotide diversity in S7 sequences

Nucleotide or amino acid sequence alignments among these 111 viral isolates from 2013 and 2014 were performed using the MegAlign program in DNAStar5.01 software (Madison, USA) [2, 44] set to default settings. The nucleotide sequences for S7 across these 111 viral isolates were aligned using MEGA 6.06 [45]. Sliding-window analyses of nucleotide diversity (π) in S7 sequences was performed using a 200-bp window in 100-bp steps with TASSEL 3.0 software [46]. Nucleotide diversities for S7 sequences were calculated for these isolates either grouped by geographic location, host, and year, or for all isolates combined.

### Detection of genetic recombination within and phylogenetic analyses of S7 sequences

Nucleotide and amino acid sequences were aligned using CLUSTAL W in MEGA 6.06 with default settings [45]. Possible recombination sites within S7 sequences were examined using the software RDP 4.22 with the RDP, GENECONV, BOOTSCAN, Maximum Chi SQUARE (MAXCHI), CHIMAERA, Sister Scanning (SISCAN), and 3Seq algorithms in the default configurations, except that the ‘linear sequence’ and ‘disentangling overlapping signals’ options were selected [47]. Recombination events were validated only if they were detected by more than two methods. The default parameter for the number of simulated datasets was 100 and the *P*-value cutoff was 0.05. Phylogenetic trees were constructed using the neighbor-joining (NJ) method in MEGA 6.06 software [45] for the S7 sequences from these 111 isolates. The number of bootstrap replicates was set to 1000. Only bootstrap values greater than 50% are shown.

### Detection of selection pressure on S7 nucleotide sequences

The *Ka/Ks* ratio was used to estimate the level of selection pressure on S7, where *Ka* is the average number of nonsynonymous substitutions per nonsynonymous site and *Ks* is the average number of synonymous substitutions per synonymous site. The average values of *Ka* and *Ks* were calculated using MEGA 6.06 software [45] according to the methods described in previous studies [48, 49]. When the *Ka/Ks* ratio is greater than one, the gene is considered to be under positive or diversifying selection. If the *Ka/Ks* ratio is one, selection is neutral. However, if the *Ka/Ks* ratio is less than one, the gene is under negative or purifying selection.

Tajima’s D, Fu & Li’s D, Fu & Li’s F statistical tests, and haplotype diversity were estimated using the software DnaSP 5.0 [50]. Tajima’s D [51], Fu and Li’s D, and Fu & Li’s F tests [52] hypothesize all mutations to be selectively neutral. The frequencies and numbers of haplotypes indicate the haplotype diversity in the population.

### Estimation of genetic differentiation and gene flow

To detect genetic differentiation between different subpopulations, three permutation-based statistical tests, Ks*, Z (the rank statistic), and Snn (the nearest-neighbor statistic), were performed. Because these three tests can powerfully detect genetic differentiation, they are particularly effective for datasets in which mutation rates are high and sample size is small [53, 54]. The level of gene flow between subpopulations was measured by estimating *Fst* (the component of genetic variation between populations or the normalized variation in allele frequencies among populations) and *Nm* (the product of the effective size of each population [N] and the rate of migration among populations [m]) [55]. *Fst* ranges from zero to one for undifferentiated to fully differentiated populations, respectively. An absolute value of *Fst* of greater than 0.33 normally suggests that infrequent gene flow has taken place. Genetic drift that can result in substantial local differentiation can be indicated if the value of *Nm* is less than one, but not if the value of *Nm* is greater than one [56]. The statistical tests for genetic differentiation and estimation of *Fst* were performed using DnaSP 5.10 [50].

## Results

### Nucleotide content and the relationship between *Nc* and GC3

The G+C contents for S7-1 and S7-2 were 35.41% and 32.29%, respectively (S1A Fig). The differences in *Nc*, *CAI*, *CBI*, GC3s, and GCs values were not significant among subpopulations across these two years, two hosts, or eight geographic locations (*P* > 0.05). However, these values were significantly higher for S7-1 than for S7-2 (*P* < 0.01) (S1A Fig). S7 would thus appear to be A+U rich, with overall codon usage biased towards codons ending with A (A3s in S7-1: 32.64%; in S7-2: 29.95%) and U (U3s in S7-1: 44.18%; in S7-2: 46.06%) (S1B Fig). In general, no significant difference was found in codon usage bias among subpopulations, delineated as years, hosts, or geographic locations (*P* > 0.05). However, all parameters for S7-1 were significantly higher than those for S7-2 (*P* < 0.01).

**Fig 1:**
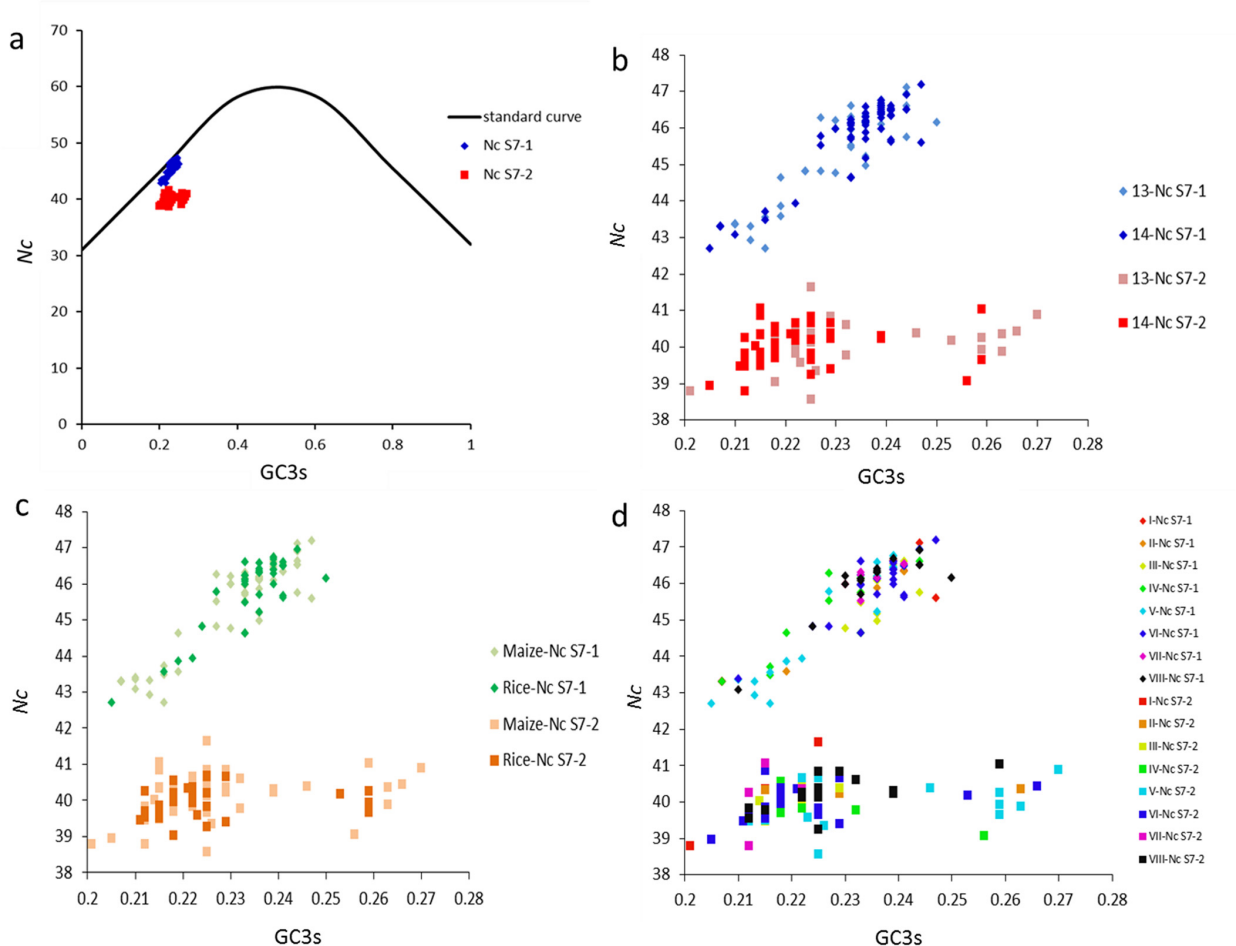
Distribution of effective number of codons (*Nc*) and GC3s in S7-1 and S7-2. (a) Distribution of *Nc* and GC3s in S7-1 and S7-2. The solid line (shown in black) indicates the standard *Nc* value if the codon bias is only due to GC3s. (b) Distribution of *Nc* and GC3s in S7-1 and S7-2 in data from two years. (c) Distribution of *Nc* and GC3s in S7-1 and S7-2 in two hosts. (d) Distribution of *Nc* and GC3s in S7-1 and S7-2 in eight geographic locations.

*Nc* plots (a plot of *Nc* versus GC3s) were used to understand the relationship between nucleotide composition and codon bias in S7-1 and S7-2 (Fig 1A). *Nc* should fall on a continuous curve between *Nc* and GC3s if GC3s is the only determinant of *Nc*. The *Nc* values for S7-1 ranged from 42 to 47 and those for S7-2 ranged from 38 to 41, indicating that there are very significant differences in codon bias between S7-1 and S7-2 (*P* < 0.01). The relationships between nucleotide composition and codon bias for both S7-1 and S7-2 are independent of years (Fig 1B), hosts (Fig 1C), and geographical locations (Fig 1D). A small number of points lie on the standard curve towards GC-poor regions in the *Nc* plot for S7-1, but no points lie on the standard curve in *Nc* plot for S7-2. However, most of the points with low *Nc* values lie below the standard curve (Fig 1A), which suggests that S7-1 and S7-2 have additional codon usage bias independent of GC3s. In fact, points for S7-2 mostly lie far away from the standard curve in comparison with those for S7-1, which indicates that mutational bias had a weaker effect on codon usage variation in S7-2 than in S7-1.

### Correspondence analysis of relative synonymous codon usage and optimal codons

**Fig 2:**
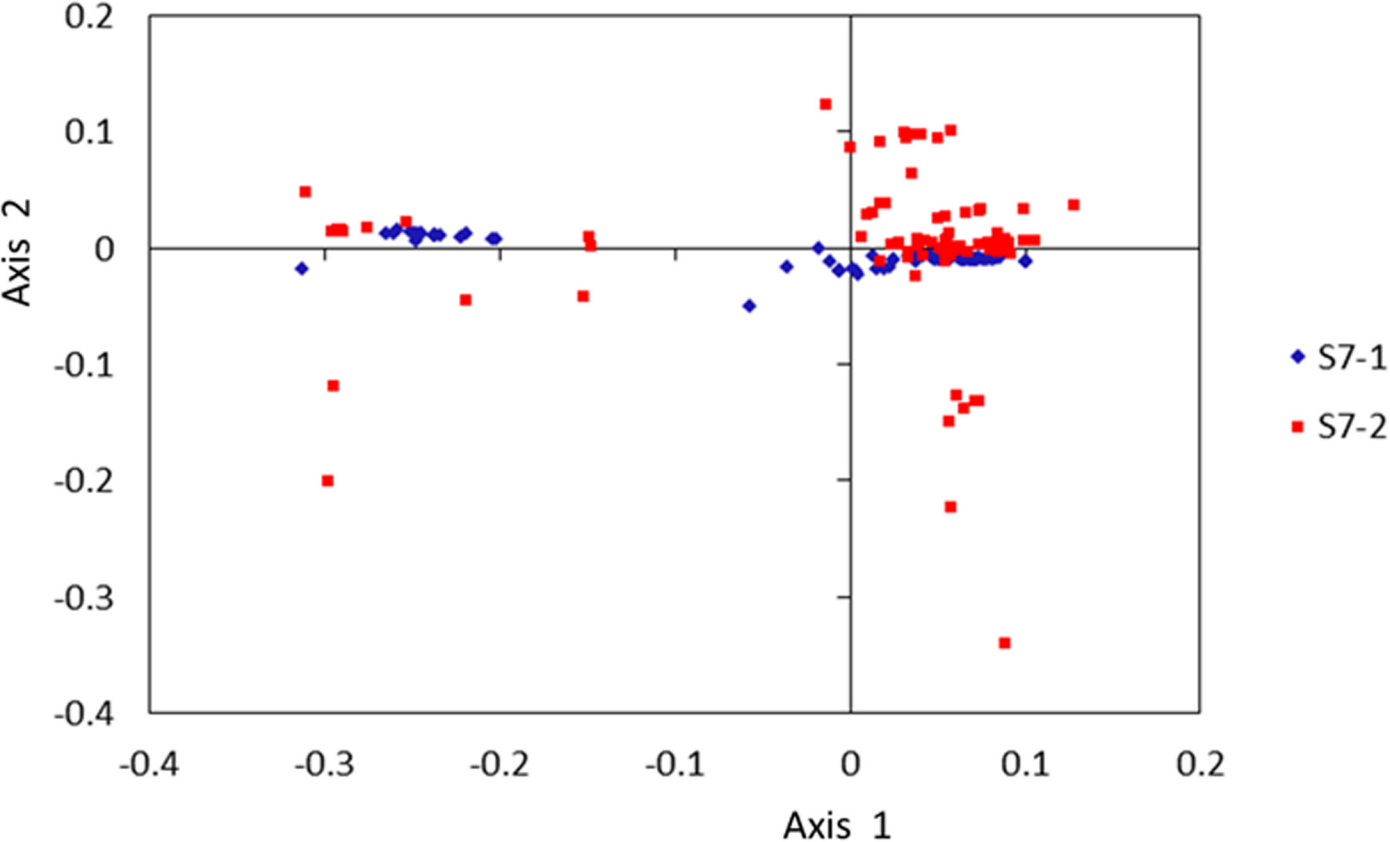
Correspondence analysis for S7-1 and S7-2 along the first and second axis. Blue diamonds represent correspondence analysis of S7-1; red squares represent correspondence analysis for S7-2.

Further evidence that mutational bias and other factors are responsible for codon usage variation in S7-1 and S7-2 came from correspondence analysis (CA) of the *RSCU* values for the two ORFs. The first two major axes explain fractions of the total variation (37.76% and 14.60% in S7-1; 38.96% and 9.64% in S7-2), and the next two axes account for 12.78% and 10.54% of the total variation in S7-1 and for 9.18% and 8.08% of the total variation in S7-2, respectively. The first and second axes for S7-1 and S7-2 were clustered in the plot (Fig 2); however, the majority of data for S7-1 and S7-2 do not cluster completely. S7-1 was scattered around the first axis, S7-2 concentrated mostly in a region located at the first quadrant of the two axes. However, the difference between S7-1 and S7-2 in this analysis was not significant (*P* > 0.05).

To detect correlations along the first two major axes for both *CAI* an*d Nc*, correlation coefficients were calculated among values of these parameters. The separation of codons on the first axis appeared to be largely due to differences in the frequencies of codons that end with G/C or A/U. The S7-1 on axis one were strongly correlated with the C3s value (*r* = 0.9560, *P* < 0.0001) and *Nc* (*r* = 0.9234, *P* < 0.0001), and significantly negatively correlated with the U3s (*r* = -0.9516, *P* < 0.0001) and G3s (*r* = -0.8720, *P* < 0.0001) values (Table 1). The S7-2 on axis one were strongly correlated with the GC3s value (*r* = 0.9241, *P* < 0.0001) and *CAI* (*r* = 0.7650, *P* < 0.0001), and significantly negatively correlated with the A3s (*r* = -0.9214, *P* < 0.0001) and GC (*r* = -0.8919, *P* < 0.0001) values (Table 1). For S7-2, values of *CAI* were significantly correlated or negatively correlated with *Nc* and certain codons (GC3s, GC, C3s, A3s, G3s) (|*r*| > 0.7, *P* < 0.0001) (Table 1). But the value of *CAI* in S7-1 was uncorrelated with *Nc* or other parameters.

**Table 1:** Correlation coefficients between the positions of S7-1 and S7-2 along the major axes and the pattern parameters of codon usage.

		Axis1	Axis2	*CAI*	*Nc*	GC3s	GC	U3s	C3s	A3s	G3s
S7-1	Axis1	1	0	0.1067	0.9234***	0.8603***	0.7994***	-0.9516	0.9560***	0.8989***	-0.8720***
	Axis2		1	0.0011	0.0248	0.0068	0.0561	0.0146	-0.0108	-0.0613	0.0427
	*CAI*			1	0.034	0.2853	0.11	-0.1793	0.1884*	-0.0211	0.0593
	*Nc*				1	0.9048***	0.9198***	-0.9485***	0.9338***	0.8029****	-0.7382***
	GC3s					1	0.9203***	-0.9528**	0.9517***	0.6646***	-0.5999***
	GC						1	-0.8922**	0.8838***	0.6262***	-0.5802***
	U3s							1	-0.9942***	-0.8585***	0.7997***
	C3s								1	0.8463***	-0.8159***
	A3s									1	-0.9571***
	G3s										1
S7-2	Axis1	1	0	0.7650***	-0.8377***	0.9241***	-0.8919***	-0.0978	0.0212	-0.9214***	-0.6581***
	Axis2		1	0.0767	-0.1819	0.0149	0.1298	0.0102	-0.2886*	-0.045	-0.0974
	*CAI*			1	-0.9201***	0.7314***	-0.7420***	-0.1844	-0.3806***	-0.9059***	-0.8621***
	*Nc*				1	-0.8447***	0.7278***	0.3223	0.2259*	0.9453***	0.8260***
	GC3s					1	-0.9283***	-0.2427	-0.0144	-0.9484***	-0.7524***
	GC						1	0.0879	0.0864	0.9104***	0.7145***
	U3s							1	-0.0017	0.246	0.3562***
	C3s								1	0.184	0.4582***
	A3s									1	0.8477***
	G3s										1

*, 0.01 < *P* < 0.05

**, 0.001 < *P* < 0.01

***P < 0.001.

To determine the optimal codons used in S7-1 and S7-2, the average *RSCU* values in high- and low-expression datasets were determined (S3 Table). Six codons were identified as the optimal codons in S7-1 and S7-2, according to the Chi-square test. Most optimal codons ended with G (41.67%) or U (33.33%), indicating that codon usage in RBSDV-S7 was biased towards synonymous codons ending with G or U.

### Nucleotide diversity across S7 in 111 viral isolates

In the present study, 76 maize isolates with typical rough dwarf disease symptoms and 35 rice isolates with typical black-streaked dwarf disease symptoms were collected from eight locations in 2013 and 2014 (S1 Table). A total of 486 nucleotide mutation sites, including 194 singleton variable sites and 292 parsimony-informative sites, were detected among the S7 sequences across these 111 viral isolates, with an average of one mutation site per five base pairs. Fourteen amino acid changes were detected in P7-1, with an average of one mutation site per 26 amino acids, and 69 amino acid changes were detected in P7-2, with an average of one mutation site per four or five amino acids.

**Fig 3:**
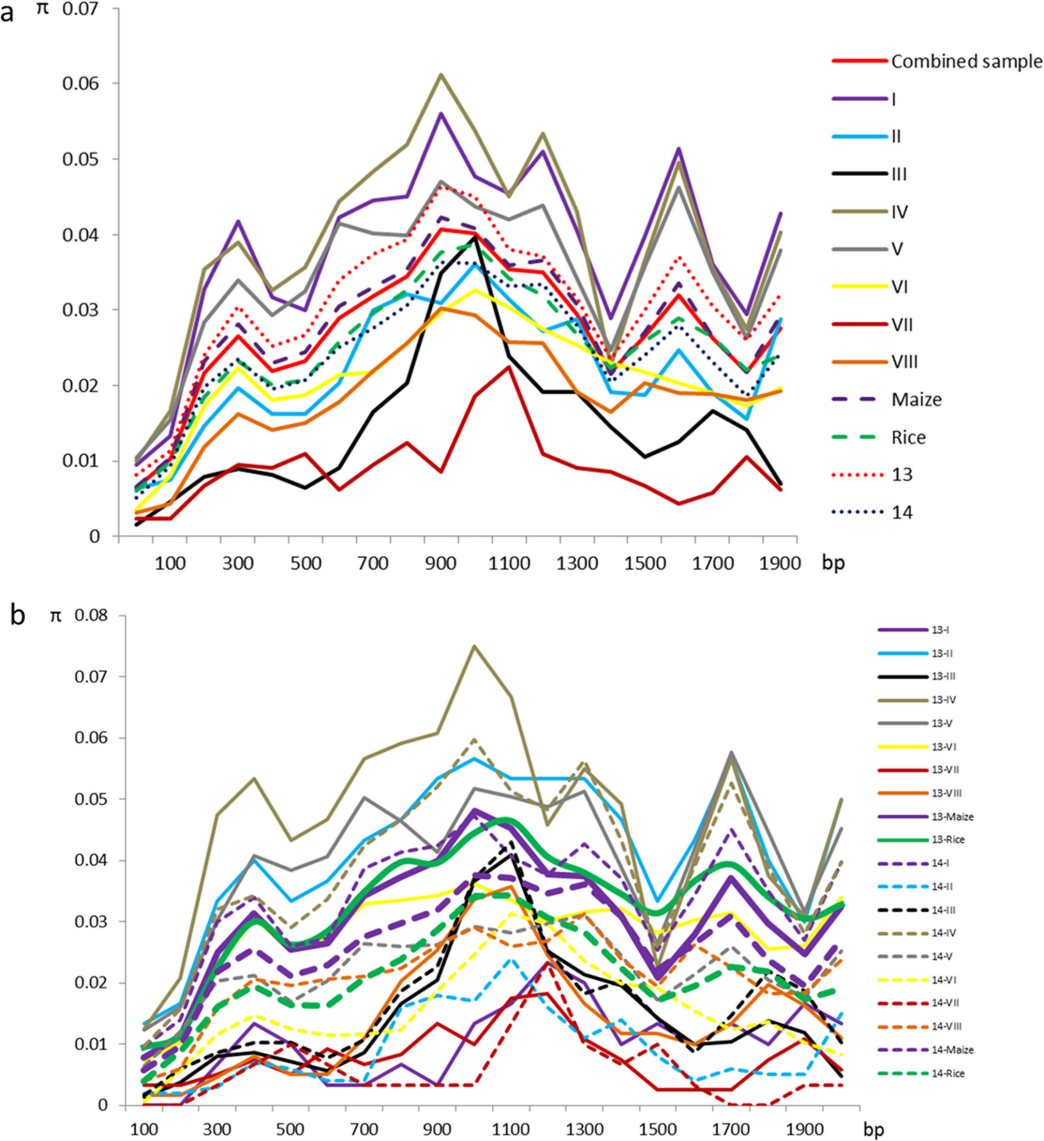
Sliding-window analysis of the nucleotide diversity in S7 sequences across Chinese isolates. (a) Sliding-window analysis of nucleotide diversity in S7 sequences calculated using a 200-bp window and 100-bp steps including combined isolates, or 111 individual Chinese isolates. I, sampled from Beijing; II, sampled from Tangshan, Hebei Province; III, sampled from Baoding, Hebei Province; IV, sampled from Jinan, Shandong Province; V, sampled from Jining, Shandong Province; VI, sampled from Zhengzhou, Henan Province; VII, sampled from Yancheng, Jiangsu Province; VIII, sampled from Nanjing, Jiangsu Province. Maize, isolates from maize hosts from eight geographic locations; Rice, isolates from rice hosts from four locations. (b) Sliding-window analysis of nucleotide diversity in S7 sequences calculated for data from two years.

Nucleotide diversity (π) for RBSDV S7 sequences was calculated across these 111 viral isolates from eight geographic locations in maize and rice hosts from 2013 and 2014. The nucleotide diversity of RBSDV S7 in the maize host (π = 0.0280) was higher than that in rice (π = 0.0253), but the *P*-value was not significant (*P* > 0.05). Isolates from Jinan (IV) showed the highest diversity (π = 0.0391), but those from Yancheng (VII) had the lowest level of nucleotide diversity (π = 0.0090) (*P* = 1.28 * 10^−27^) (Fig 3A). The nucleotide diversity in 2013, with a π value of 0.0307, was significantly higher than that in 2014, with a π value of 0.0244 (*P* = 0.0226). Most polymorphisms in S7 were identified in the sequence region from 800 to 1200 bp among isolates sampled in Baoding (Fig 3A). The nucleotide diversity of RBSDV S7 from the geographic locations Tangshan (π = 0.0402), Jining (π = 0.0402), and Zhengzhou (π = 0.0223), and in the rice host (π = 0.0326) in 2013 were very significantly higher than that in Tangshan (π = 0.0093), Jining (π = 0.0223), Zhengzhou (π = 0.0154), and in the rice host (π = 0.0211) in 2014 (*P* < 0.01). The nucleotide diversity of RBSDV S7 from the geographic location Beijing (π = 0.0103) in 2013 was very significant lower than that (π = 0.0337) in 2014 (*P* < 0.01). The nucleotide diversity of RBSDV S7 from geographic location Jinan (π = 0.0465) in 2013 was significantly higher than that (π = 0.0389) in 2014 (0.01 < *P* < 0.05). The nucleotide diversity at geographic location Nanjing (π = 0.0144) in 2013 was significantly higher than that (π = 0.0211) in 2014 (0.01 < *P* < 0.05). The nucleotide diversity of the other three subgroups, including Baoding, Yancheng, and maize, were not significantly different in 2013 or 2014 (Fig 3B).

### Recombination and phylogenetic analysis

One recombination event within S7 was detected in maize isolate 13IIIM-2 from Baoding using three different methods (Maxchi, Chimaera, SiSscan). The breakpoint positions were located at nucleotide (nt) 1242 in ORF S7-2 and at nt 2192 in the 3’ UTR of 13IIIM-2 within the major and minor parental sequences for isolates 13VIIM-4 and 13VR-2.

**Fig 4:**
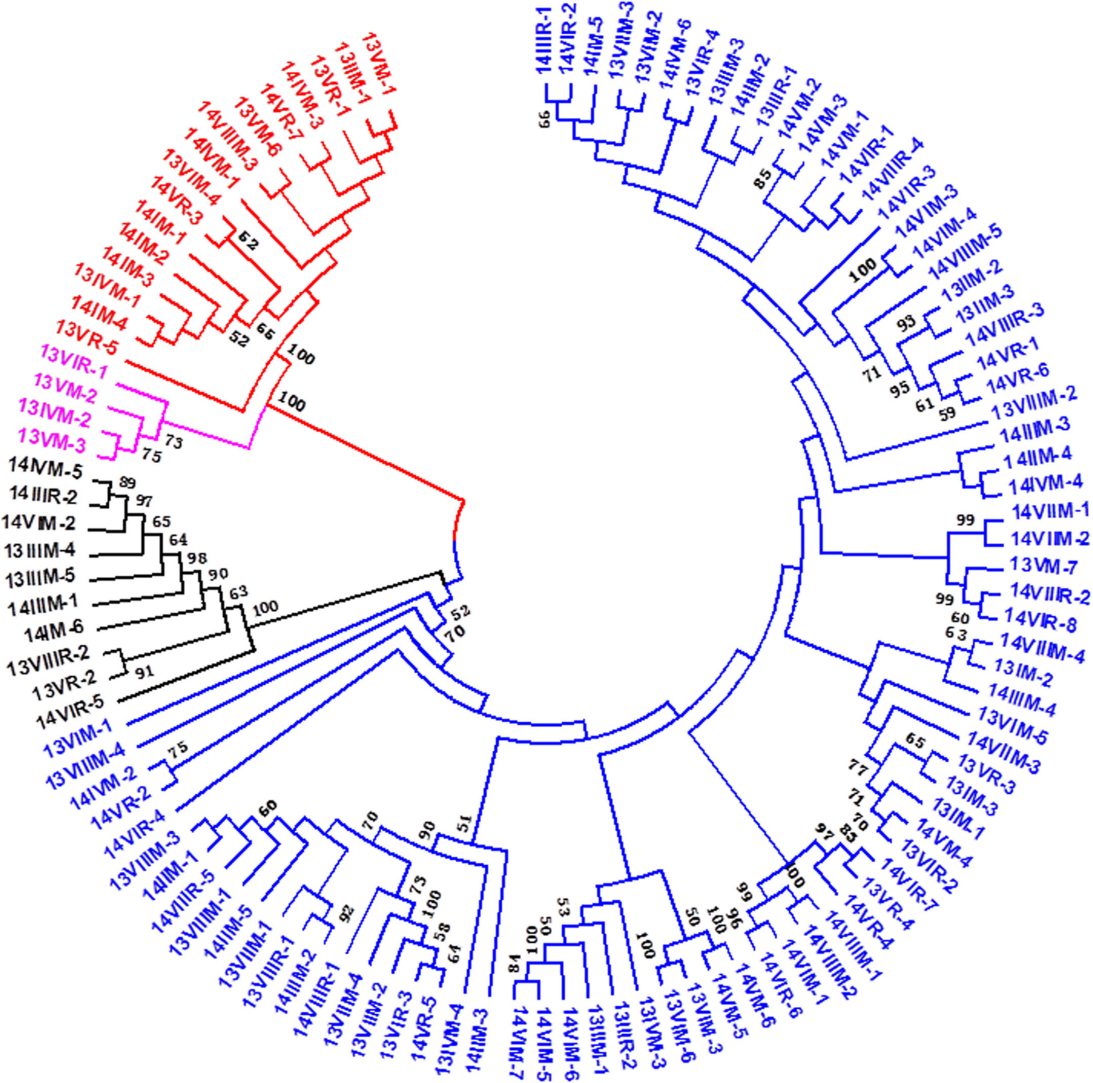
Neighbor-joining phylogenetic tree based on the nonrecombinant nucleotide sequence of S7 from different RBSDV isolates. The number of bootstrap replicates was set to 1000. Only bootstrap values > 50% are shown. Red lines represent the isolates that clustered into subgroup AI; pink lines represent the isolates that clustered into subgroup AII; black lines represent the isolates that clustered into subgroup BI; blue lines represent the isolates that clustered into subgroup BII.

A phylogenetic tree was constructed from these 110 isolate sequences to determine the evolutionary relationships among these RBSDV S7 isolates. The recombinant in the present study was not included, because the phylogenetic algorithm we used cannot accommodate recombinants (Fig 4). Based on S7 sequences, these 110 isolates could be classified into two main groups, designated A and B, that were independent of year, host, and geographical origin (Fig 4). Both groups A and B could be further clustered into two subgroups (groups AI and AII; and BI and BII). Subgroup AI included seven isolates from 2013 and nine isolates from 2014; subgroup AII included four isolates from 2013; subgroup BI included four isolates from 2013 and six isolates from 2014; subgroup BII included 31 isolates from 2013 and 49 isolates from 2014.

### Selection pressure and neutrality tests

To analyze possible selection pressure on RBSDV S7, the ratios of nonsynonymous to synonymous sites (*Ka/Ks*) were calculated for maize and rice hosts from eight geographic locations in 2013 and 2014 (Table 2). The *Ka/Ks* ratios for S7-1 and S7-2 suggest that both S7-1 and S7-2 were under negative and purifying selection (Table 2). There was no significant difference in *Ka/Ks* ratios for S7-1 or S7-2 between 2013 and 2014, with S7-1 values of 0.0181 and 0.0177, and S7-2 values of 0.0510 and 0.0569, respectively. And there were no significant differences in *Ka/Ks* ratios for S7-1 and S7-2 between hosts or between geographic locations. However, *Ka/Ks* ratios for S7-1, which ranged from 0.0147 to 0.0370, were significantly lower than those for S7-2, which ranged from 0.0328 to 0.0702 (*P* < 0.01). This result suggests that S7-1 and S7-2 each experienced different levels of selection, and that selection pressure on S7-1 was greater than that on S7-2.

**Table 2:** Nonsynonymous-to-synonymous substitution ratio for S7-1 and S7-2 sequences from RBSDV.

	S7-1			S7-2		
	*Ka*	*Ks*	*Ka/Ks*	*Ka*	*Ks*	*Ka/Ks*
Total	0.0022	0.1231	0.0179	0.0067	0.1247	0.0537
2013	0.0026	0.1435	0.0181	0.0072	0.1413	0.0510
2014	0.0019	0.1073	0.0177	0.0064	0.1125	0.0569
Maize	0.0024	0.1291	0.0186	0.0069	0.1289	0.0535
Rice	0.0017	0.1111	0.0153	0.0064	0.1173	0.0546
I	0.0031	0.1760	0.0176	0.0098	0.1854	0.0529
II	0.0019	0.0959	0.0198	0.0068	0.1030	0.0660
III	0.0021	0.0567	0.0370	0.0036	0.0513	0.0702
IV	0.0032	0.1935	0.0165	0.0090	0.1788	0.0503
V	0.0024	0.1629	0.0147	0.0085	0.1650	0.0515
VI	0.0017	0.0908	0.0187	0.0057	0.0950	0.0600
VII	0.0007	0.0343	0.0204	0.0012	0.0366	0.0328
VIII	0.0014	0.0766	0.0183	0.0046	0.0879	0.0523

Values for Tajima’s D, Fu and Li’s D, and Fu and Li’s F, as well as haplotype, were evaluated using DnaSP version 5.10 (Table 3). The values for Tajima’s D, Fu and Li’s D, and Fu and Li’s F were all negative for year, host, and geographic location except in locations I and IV. The *P*-values for Tajima’s D, and Fu and Li’s D, and Li’s D and F were less than 0.01 in the entire population of 111 isolates and less than 0.05 in the maize, location VI, and location VIII subpopulations. This result suggests that the RBSDV populations were expanding (*P* < 0.01). The maize, location VI, and location VIII subpopulations were in a state of significant expansion (*P* < 0.05). The other subpopulations were also expanding, but not significantly. The values of haplotype diversity for S7 ranged from 0.8330 to 1.000 in different subpopulations. Such high values for haplotype diversity also indicate that the RBSDV populations were expanding.

**Table 3:** Neutrality tests and haplotypes of S7-1 and S7-2 in subpopulations.

Subpopulation		Tajima's D	Fu and Li's D	Fu and Li's F	Haplotype diversity
Combined sample		-1.5102	-3.4787**	-3.1068**	0.9980±0.0020
Year	2014	-1.3653	-1.9178	-2.0345	0.9950±0.0040
	2013	-1.0203	-2.1922	-2.0957	0.9980±0.0050
Host	Maize	-1.2512	-2.5333*	-2.3991*	0.9960±0.0030
	Rice	-1.3894	-1.6986	-1.8911	0.9980±0.0070
Geographic location	I	1.0356	0.3773	0.6040	0.8330±0.1270
	II	-1.5566	-1.6362	-1.8070	0.9640±0.0770
	III	-0.4384	-1.1045	-1.0593	1.0000±0.0300
	IV	0.4170	-0.0950	0.0378	1.0000±0.0450
	V	-0.0291	-0.7090	-0.5810	0.9960±0.0002
	VI	-1.8487*	-1.2549	-1.7028	0.9930±0.0130
	VII	-0.3289	-0.3034	-0.3421	0.9520±0.0960
	VIII	-1.9700*	-2.3057	-2.5564*	1.0000±0.0220

*, 0.01 < *P* < 0.05

**, 0.001 < *P* < 0.01.

### Genetic differentiation and gene flow between subpopulations

In the present study, genetic differentiation and gene flow between RBSDV subpopulations, including years, hosts, and geographic locations, were analyzed. The *P*-values for Ks*, Z, and Snn calculated from RBSDV S7 subpopulations derived from 2013 or 2014 and the subpopulations derived from maize or rice were greater than 0.05. These results suggest that genetic differentiation was not significant between subpopulations defined as years or hosts (Table 4). However, genetic differentiation of six particular groups of subpopulations reached significant or very significant levels (Table 4). These six groups were derived from the combinations of locations I and III, I and VII, I and VIII, III and V, III and VII, and IV and VII.

**Table 4:** Genetic differentiation and gene flow between subpopulations of RBSDV-S7.

Subpopulation	Ks*	(*P*-value)	Z	(*P*-value)	Snn	(*P*-value)	*F*_*ST*_	*Nm*
2013–2014	3.6750	0.2460	3046.6826	0.2710	0.5886	0.0790	0.0039	65.12
Maize-Rice	3.6807	0.4910	3060.8268	0.6960	0.6171	0.1720	-0.0065	-38.49
I–II	3.5227	0.056	64.7369	0.1760	0.7451	0.0140*	0.1177	1.87
I–III	3.3783	0.0070**	101.5366	0.00100*	0.7879	0.0050*	0.2387	0.80
I–IV	3.8881	0.3280	88.2189	0.9320	0.6316	0.0820	-0.0793	-3.40
I–V	3.7973	0.1220	250.3742	0.6340	0.7563	0.0310*	-0.0284	-9.05
I–VI	3.5592	0.0060**	261.9209	0.0130*	0.6912	0.1800	0.1571	1.34
I–VII	3.2813	0.0100*	52.5337	0.0210*	0.8333	0.0030**	0.3069	0.56
I–VIII	3.4757	0.0030*	136.2528	0.0150*	0.7600	0.0160*	0.1863	1.09
II–III	3.2745	0.0560	99.8375	0.1130	0.7619	0.0220*	0.0683	3.41
II–IV	3.7984	0.1710	75.2973	0.2720	0.6019	0.1770	0.0612	3.83
II–V	3.7474	0.2360	231.4365	0.3290	0.8011	0.150*	0.0404	5.94
II–VI	3.5045	0.1650	263.9521	0.4330	0.7879	0.0400*	-0.0259	-9.92
II–VII	3.1120	0.1170	52.9654	0.2520	0.6600	0.1150	0.0169	14.51
II–VIII	3.3923	0.4980	139.9692	0.7890	0.5208	0.5970	-0.0498	-5.27
III–IV	3.5891	0.0350*	116.5681	0.0440*	0.6522	0.1030	0.1764	1.17
III–V	3.6311	0.0090**	299.5385	0.0140*	0.7917	0.0030**	0.1774	1.16
III–VI	3.4308	0.0660	346.9428	0.1740	0.5790	0.3800	0.0560	4.22
III–VII	3.0779	0.0190*	88.0398	0.0350*	0.8500	0.0080**	0.1838	1.11
III–VIII	3.3145	0.0450*	195.5408	0.0160*	0.6379	0.0790	0.0682	3.42
IV–V	3.9209	0.6920	267.7405	0.7870	0.4546	0.8860	-0.0531	-4.96
IV–VI	3.6825	0.0150*	286.5140	0.0870	0.5857	0.5270	0.0997	2.26
IV–VII	3.5968	0.0210*	62.6031	0.0430*	0.7745	0.0210*	0.2408	0.79
IV–VIII	3.6533	0.0300*	153.9128	0.0390*	0.6026	0.1910	0.1252	1.75
V–VI	3.6855	0.0270*	551.3970	0.0600	0.5833	0.1550	0.0818	2.81
V–VII	3.6446	0.0480*	211.2653	0.0920	0.8833	0.0020*	0.2156	0.91
V–VIII	3.6679	0.0630	358.9741	0.0690	0.6581	0.0670	0.1044	2.14
VI–VII	3.4004	0.0890	247.4075	0.3700	0.8594	0.0120*	0.0591	3.98
VI–VIII	3.4818	0.1900	409.3579	0.0750	0.5854	0.2600	-0.0085	-29.61
VII–VIII	3.2330	0.1990	128.2852	0.7740	0.7029	0.1100	0.0187	13.11

*, 0.01 < *P* < 0.05

**, 0.001 < *P* < 0.01.

The absolute values of *Fst* for subpopulations based on years, hosts, and geographic locations were less than 0.33, indicating gene flow between subpopulations of RBSDV (Table 4). Gene flow was the most frequent across years, because the absolute *Fst* values for subpopulations comprised of 2013 and 2014 were the smallest. The absolute values of *Nm* for subpopulations comprised of 2013 and 2014, maize and rice hosts, and 24 groups based on geographic locations (except for combined locations I + III, I + VII, IV + VII, and V+VII) were greater than one (Table 4). This result suggests that gene flow occurred between years or parts of geographic locations. The absolute values of *Nm* were greater than four in some subpopulations, such as 2013 and 2014, hosts maize and rice, and combined geographic locations I and V, II and V, II and VI, II and VII, II and VIII, III and VI, IV and V, VI and VIII, and VII and VIII, suggesting that gene flow occurred frequently among these subpopulations.

## Discussion

MRDD is a serious viral plant disease in the Yellow and Huai River summer maize-growing region of China, in which winter wheat is also grown [57–59]. Maize and rice are infested naturally by the small brown planthopper (SBPH) viral vector that overwinters on winter wheat [60, 61]. The SBPH also migrates between regions in China and infects maize or rice [1, 62], so variation in the virus could occur during migration and reproduction of this vector. The genetic diversity of the virus might be supported by its frequent transmission by the SBPH vector in maize and rice hosts in these eight geographic locations in 2013 and 2014. In the present study, the levels of nucleotide diversity observed in these isolates were similar in maize and rice, independent of geographic locations or years. However, the differences in observed nucleotide diversity among years or parts of geographic locations reached significant levels (*P* < 0.05). So it is possible that the distinct levels of nucleotide diversity in these two years and eight geographic locations may be greater than that in the two hosts.

High levels of adaptation of codon usage have been reported for several viruses including those in the family *Flaviviridae*, which infect humans, and in other viruses that infect bacteria and humans [63, 64]. A detailed comparative analysis was performed to evaluate the level of codon usage bias occurring in RBSDV S7 sequences. In general, RBSDV S7 exhibits a low degree of codon usage bias (average *Nc*, S7-1: 45.63, S7-2: 39.96), thus mutational bias is likely to be the major force driving codon usage bias in RBSDV S7. The nucleotide composition of these genes provided evidence of mutation as the major factor influencing the codon usage bias between S7-1 and S7-2 but not towards convergence. This result is consistent with previous reports showing that mutational bias is the major force that affects codon usage in other viruses [20, 65, 66]. Previous studies have shown that protein secondary structure and genomic architecture also influence codon usage bias in plant viruses [67]. Combining information from the conserved sequence of RBSDV S7 and its codon usage pattern, an RNA interference (RNAi) vector could be constructed to use to transform maize for disease resistance.

Previous studies have shown that the RBSDV population in China can be organized into three groups based on S8 sequences [1], or into two groups based on S9 [32] and S10 sequences [1, 2], regardless of host or geographic origin. In the present study, 111 Chinese S7 isolates also clustered into two groups without regard to host or geographic location. This result also conforms with the results of a previous study on RBSDV S9 [32]. However, in the present study, years influenced the grouping to some degree. Within subgroup A, AI was widely distributed, while AII was comprised of the isolates from only 2013. Some isolates from 2014 clustered into subgroup BII. These results provided direct evidence of the irrelevance of hosts or different geographic locations but the relevance of years in regard to genetic variation among RBSDV isolates. Correspondence analysis of relative synonymous codon usage revealed a relationship between the phylogeny and the first and second axes of S7-1 and S7-2. These results suggest that the phylogenetic clusters are correlated with the values for *CAI*, *Nc*, GC3s, GC, AC3s, and G3s.

Population genetic structure is a significant aspect influencing the evolution of plant viruses and several studies of the genetic structure of plant virus populations have been reported. However, genetic structure had rarely been studied in S7 sequences from RBSDV or other segments of similar viruses harboring two ORFs. Frequent gene flow events were detected between the subpopulations comprised of two years, two hosts, and most of the geographic locations analysed, especially among year and host subpopulations in China. These results suggest that gene flow between years was more frequent than that between hosts, and that gene flow between geographic locations was the lowest. Because only S7 sequences were investigated in the present study, more evidence from other segments of RBSDV should be gathered to verify this hypothesis.

In conclusion, the genetic structure and codon usage bias of RBSDV S7 sequences were determined for 111 Chinese isolates from maize and rice hosts obtained from eight geographic locations in 2013 and 2014. Genetic variation and genetic structure were analysed for the RBSDV S7 dsRNA sequence that is comprised of two ORFs. Further, the present study represents the first time that codon usage bias in RBSDV has been analysed. These results should help elucidate the evolution of this virus and promote further exploration of the relationship between this virus and its hosts.

## Supporting Information

S1 Fig Basic characteristics of codon usage in S7-1 and S7-2. (a) Values for Nc, CAI, CBI, GC3s, and GC in S7-1 and S7-2 in data for two years, two hosts, and eight geographic locations are shown. (b) Values for G3s, A3s, C3s, and U3s in S7-1 and S7-2 in data for two years, two hosts, and eight geographic locations are shown.
(TIF)

S1 Table Information regarding RBSDV isolates described in the present study. (DOCX)

S2 Table Specific primers used for amplification and sequencing of S7 sequences. (DOCX)

S3 Table Relative synonymous codon usage (*RSCU*) values for each codon in S7-1 and S7-2. (DOCX)
