# Supplementary figures and images for: Molecular Genetic Analysis and Evolution of Segment 7 in Rice Black-Streaked Dwarf Virus in China
Source: PLoS One. 2015 Jun 29;10(6):e0131410. doi: 10.1371/journal.pone.0131410 (PMC4488072; doi:10.1371/journal.pone.0131410)

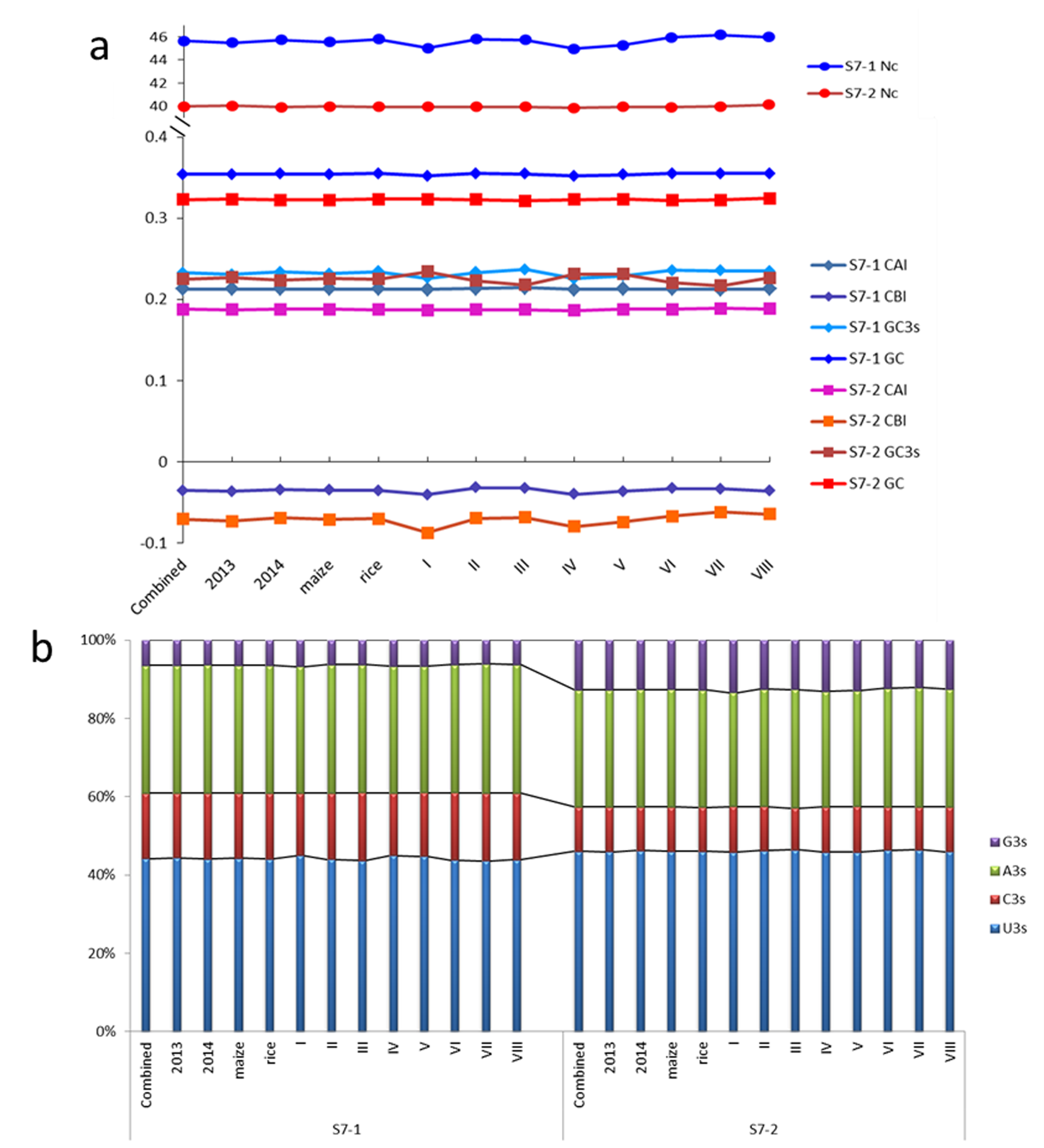

Supplement: S1 Fig — (a) Values for Nc, CAI, CBI, GC3s, and GC in S7-1 and S7-2 in data for two years, two hosts, and eight geographic locations are shown. (b) Values for G3s, A3s, C3s, and U3s in S7-1 and S7-2 in data for two years, two hosts, and eight geographic locations are shown. (TIF) [file pone.0131410.s001.tif]
