# Supplementary material for: Molecular Genetic Analysis and Evolution of Segment 7 in Rice Black-Streaked Dwarf Virus in China
Source: PLoS One. 2015 Jun 29;10(6):e0131410. doi: 10.1371/journal.pone.0131410 (PMC4488072; doi:10.1371/journal.pone.0131410)
Supplement: S1 Table — (DOCX) [file pone.0131410.s002.docx]

**S1 Table Information regarding RBSDV isolates described in the present study**

| Code | Location | Host | Sampling day | Latitude | Longitude |
| --- | --- | --- | --- | --- | --- |
| 13IM-1 | Beijing | maize | 08/25/2013 | 39°57′ | 116°19′ |
| 13IM-2 | Beijing | maize | 08/25/2013 | 39°57′ | 116°19′ |
| 13IM-3 | Beijing | maize | 08/25/2013 | 39°57′ | 116°19′ |
| 13IIM-1 | Tangshan, Hebei | maize | 08/05/2013 | 39°37′ | 118°10′ |
| 13IIM-2 | Tangshan, Hebei | maize | 08/05/2013 | 39°37′ | 118°10′ |
| 13IIM-3 | Tangshan, Hebei | maize | 08/05/2013 | 39°37′ | 118°10′ |
| 13IIIM-1 | Baoding, Heibei | maize | 08/06/2013 | 38°52′ | 116°02′ |
| 13IIIM-2 | Baoding, Heibei | maize | 08/06/2013 | 38°52′ | 116°02′ |
| 13IIIM-3 | Baoding, Heibei | maize | 08/06/2013 | 38°52′ | 116°02′ |
| 13IIIM-4 | Baoding, Heibei | maize | 08/06/2013 | 38°52′ | 116°02′ |
| 13IIIM-5 | Baoding, Heibei | maize | 08/06/2013 | 38°52′ | 116°02′ |
| 13IIIR-1 | Baoding, Heibei | rice | 08/06/2013 | 38°52′ | 116°02′ |
| 13IIIR-2 | Baoding, Heibei | rice | 08/06/2013 | 38°52′ | 116°02′ |
| 13IVM-1 | Jinan, Shandong | maize | 07/24/2013 | 36°39′ | 117°06′ |
| 13IVM-2 | Jinan, Shandong | maize | 07/24/2013 | 36°39′ | 117°06′ |
| 13IVM-3 | Jinan, Shandong | maize | 07/24/2013 | 36°39′ | 117°06′ |
| 13IVM-4 | Jinan, Shandong | maize | 07/24/2013 | 36°39′ | 117°06′ |
| 13VM-1 | Jining, Shandong | maize | 07/24/2013 | 35°24′ | 116°34′ |
| 13VM-2 | Jining, Shandong | maize | 07/24/2013 | 35°24′ | 116°34′ |
| 13VM-3 | Jining, Shandong | maize | 07/24/2013 | 35°24′ | 116°34′ |
| 13VM-6 | Jining, Shandong | maize | 07/24/2013 | 35°24′ | 116°34′ |
| 13VM-7 | Jining, Shandong | maize | 07/24/2013 | 35°24′ | 116°34′ |
| 13VR-1 | Jining, Shandong | rice | 07/24/2013 | 35°24′ | 116°34′ |
| 13VR-2 | Jining, Shandong | rice | 07/24/2013 | 35°24′ | 116°34′ |
| 13VR-3 | Jining, Shandong | rice | 07/24/2013 | 35°24′ | 116°34′ |
| 13VR-4 | Jining, Shandong | rice | 07/24/2013 | 35°24′ | 116°34′ |
| 13VR-5 | Jining, Shandong | rice | 07/24/2013 | 35°24′ | 116°34′ |
| 13VIM-1 | Zhengzhou, Henan | maize | 08/21/2013 | 34°43′ | 113°37′ |
| 13VIM-2 | Zhengzhou, Henan | maize | 08/21/2013 | 34°43′ | 113°37′ |
| 13VIM-3 | Zhengzhou, Henan | maize | 08/21/2013 | 34°43′ | 113°37′ |
| 13VIM-4 | Zhengzhou, Henan | maize | 08/21/2013 | 34°43′ | 113°37′ |
| 13VIM-5 | Zhengzhou, Henan | maize | 08/21/2013 | 34°43′ | 113°37′ |
| 13VIM-6 | Zhengzhou, Henan | maize | 08/21/2013 | 34°43′ | 113°37′ |
| 13VIR-1 | Zhengzhou, Henan | rice | 08/21/2013 | 34°43′ | 113°37′ |
| 13VIR-2 | Zhengzhou, Henan | rice | 08/21/2013 | 34°43′ | 113°37′ |
| 13VIR-3 | Zhengzhou, Henan | rice | 08/21/2013 | 34°43′ | 113°37′ |
| 13VIR-4 | Zhengzhou, Henan | rice | 08/21/2013 | 34°43′ | 113°37′ |
| 13VIIM-1 | Yancheng, Jiangsu | maize | 07/26/2013 | 33°20′ | 120°09′ |
| 13VIIM-2 | Yancheng, Jiangsu | maize | 07/26/2013 | 33°20′ | 120°09′ |
| 13VIIM-3 | Yancheng, Jiangsu | maize | 07/26/2013 | 33°20′ | 120°09′ |
| 13VIIM-4 | Yancheng, Jiangsu | maize | 07/26/2013 | 33°20′ | 120°09′ |
| 13VIIIM-1 | Nanjing, Jiangsu | maize | 07/26/2013 | 32°02′ | 118°52′ |
| 13VIIIM-2 | Nanjing, Jiangsu | maize | 07/26/2013 | 32°02′ | 118°52′ |
| 13VIIIM-3 | Nanjing, Jiangsu | maize | 07/26/2013 | 32°02′ | 118°52′ |
| 13VIIIM-4 | Nanjing, Jiangsu | maize | 07/26/2013 | 32°02′ | 118°52′ |
| 13VIIIR-1 | Nanjing, Jiangsu | rice | 07/26/2013 | 32°02′ | 118°52′ |
| 13VIIIR-2 | Nanjing, Jiangsu | rice | 07/26/2013 | 32°02′ | 118°52′ |
| 14IM-1 | Beijing | maize | 07/22/2014 | 39°57′ | 116°19′ |
| 14IM-2 | Beijing | maize | 07/22/2014 | 39°57′ | 116°19′ |
| 14IM-3 | Beijing | maize | 07/22/2014 | 39°57′ | 116°19′ |
| 14IM-4 | Beijing | maize | 07/22/2014 | 39°57′ | 116°19′ |
| 14IM-5 | Beijing | maize | 07/22/2014 | 39°57′ | 116°19′ |
| 14IM-6 | Beijing | maize | 07/22/2014 | 39°57′ | 116°19′ |
| 14IIM-1 | Tangshan, Hebei | maize | 07/23/2014 | 39°36′ | 118°55′ |
| 14IIM-2 | Tangshan, Hebei | maize | 07/23/2014 | 39°36′ | 118°55′ |
| 14IIM-3 | Tangshan, Hebei | maize | 07/23/2014 | 39°36′ | 118°55′ |
| 14IIM-4 | Tangshan, Hebei | maize | 07/23/2014 | 39°36′ | 118°55′ |
| 14IIM-5 | Tangshan, Hebei | maize | 07/23/2014 | 39°36′ | 118°55′ |
| 14IIIM-1 | Baoding, Heibei | maize | 07/15/2014 | 38°52′ | 116°02′ |
| 14IIIM-2 | Baoding, Heibei | maize | 07/15/2014 | 38°52′ | 116°02′ |
| 14IIIM-3 | Baoding, Heibei | maize | 07/15/2014 | 38°52′ | 116°02′ |
| 14IIIM-4 | Baoding, Heibei | maize | 07/15/2014 | 38°52′ | 116°02′ |
| 14IIIR-1 | Baoding, Heibei | rice | 07/15/2014 | 38°52′ | 116°02′ |
| 14IIIR-2 | Baoding, Heibei | rice | 07/15/2014 | 38°52′ | 116°02′ |
| 14IVM-1 | Jinan, Shandong | maize | 07/28/2014 | 36°39′ | 117°06′ |
| 14IVM-2 | Jinan, Shandong | maize | 07/28/2014 | 36°39′ | 117°06′ |
| 14IVM-3 | Jinan, Shandong | maize | 07/28/2014 | 36°39′ | 117°06′ |
| 14IVM-4 | Jinan, Shandong | maize | 07/28/2014 | 36°39′ | 117°06′ |
| 14IVM-5 | Jinan, Shandong | maize | 07/28/2014 | 36°39′ | 117°06′ |
| 14IVM-6 | Jinan, Shandong | maize | 07/28/2014 | 36°39′ | 117°06′ |
| 14VM-1 | Jining, Shandong | maize | 07/28/2014 | 35°24′ | 116°34′ |
| 14VM-2 | Jining, Shandong | maize | 07/28/2014 | 35°24′ | 116°34′ |
| 14VM-3 | Jining, Shandong | maize | 07/28/2014 | 35°24′ | 116°34′ |
| 14VM-4 | Jining, Shandong | maize | 07/28/2014 | 35°24′ | 116°34′ |
| 14VM-5 | Jining, Shandong | maize | 07/28/2014 | 35°24′ | 116°34′ |
| 14VM-6 | Jining, Shandong | maize | 07/28/2014 | 35°24′ | 116°34′ |
| 14VR-1 | Jining, Shandong | rice | 07/28/2014 | 35°24′ | 116°34′ |
| 14VR-2 | Jining, Shandong | rice | 07/28/2014 | 35°24′ | 116°34′ |
| 14VR-3 | Jining, Shandong | rice | 07/28/2014 | 35°24′ | 116°34′ |
| 14VR-4 | Jining, Shandong | rice | 07/28/2014 | 35°24′ | 116°34′ |
| 14VR-5 | Jining, Shandong | rice | 07/28/2014 | 35°24′ | 116°34′ |
| 14VR-6 | Jining, Shandong | rice | 07/28/2014 | 35°24′ | 116°34′ |
| 14VR-7 | Jining, Shandong | rice | 07/28/2014 | 35°24′ | 116°34′ |
| 14VIM-1 | Zhengzhou, Henan | maize | 08/10/2014 | 34°43′ | 113°37′ |
| 14VIM-2 | Zhengzhou, Henan | maize | 08/10/2014 | 34°43′ | 113°37′ |
| 14VIM-3 | Zhengzhou, Henan | maize | 08/10/2014 | 34°43′ | 113°37′ |
| 14VIM-4 | Zhengzhou, Henan | maize | 08/10/2014 | 34°43′ | 113°37′ |
| 14VIM-5 | Zhengzhou, Henan | maize | 08/10/2014 | 34°43′ | 113°37′ |
| 14VIM-6 | Zhengzhou, Henan | maize | 08/10/2014 | 34°43′ | 113°37′ |
| 14VIM-7 | Zhengzhou, Henan | maize | 08/10/2014 | 34°43′ | 113°37′ |
| 14VIR-1 | Zhengzhou, Henan | rice | 08/10/2014 | 34°43′ | 113°37′ |
| 14VIR-2 | Zhengzhou, Henan | rice | 08/10/2014 | 34°43′ | 113°37′ |
| 14VIR-3 | Zhengzhou, Henan | rice | 08/10/2014 | 34°43′ | 113°37′ |
| 14VIR-4 | Zhengzhou, Henan | rice | 08/10/2014 | 34°43′ | 113°37′ |
| 14VIR-5 | Zhengzhou, Henan | rice | 08/10/2014 | 34°43′ | 113°37′ |
| 14VIR-6 | Zhengzhou, Henan | rice | 08/10/2014 | 34°43′ | 113°37′ |
| 14VIR-7 | Zhengzhou, Henan | rice | 08/10/2014 | 34°43′ | 113°37′ |
| 14VIR-8 | Zhengzhou, Henan | rice | 08/10/2014 | 34°43′ | 113°37′ |
| 14VIIM-1 | Yancheng, Jiangsu | maize | 07/26/2014 | 33°20′ | 120°09′ |
| 14VIIM-2 | Yancheng, Jiangsu | maize | 07/26/2014 | 33°20′ | 120°09′ |
| 14VIIM-3 | Yancheng, Jiangsu | maize | 07/26/2014 | 33°20′ | 120°09′ |
| 14VIIIM-1 | Nanjing, Jiangsu | maize | 07/25/2014 | 32°02′ | 118°52′ |
| 14VIIIM-2 | Nanjing, Jiangsu | maize | 07/25/2014 | 32°02′ | 118°52′ |
| 14VIIIM-3 | Nanjing, Jiangsu | maize | 07/25/2014 | 32°02′ | 118°52′ |
| 14VIIIM-4 | Nanjing, Jiangsu | maize | 07/25/2014 | 32°02′ | 118°52′ |
| 14VIIIM-5 | Nanjing, Jiangsu | maize | 07/25/2014 | 32°02′ | 118°52′ |
| 14VIIIR-1 | Nanjing, Jiangsu | rice | 07/25/2014 | 32°02′ | 118°52′ |
| 14VIIIR-2 | Nanjing, Jiangsu | rice | 07/25/2014 | 32°02′ | 118°52′ |
| 14VIIIR-3 | Nanjing, Jiangsu | rice | 07/25/2014 | 32°02′ | 118°52′ |
| 14VIIIR-4 | Nanjing, Jiangsu | rice | 07/25/2014 | 32°02′ | 118°52′ |
| 14VIIIR-5 | Nanjing, Jiangsu | rice | 07/25/2014 | 32°02′ | 118°52′ |
