# Supplementary material for: Molecular Genetic Analysis and Evolution of Segment 7 in Rice Black-Streaked Dwarf Virus in China
Source: PLoS One. 2015 Jun 29;10(6):e0131410. doi: 10.1371/journal.pone.0131410 (PMC4488072; doi:10.1371/journal.pone.0131410)
Supplement: S2 Table — (DOCX) [file pone.0131410.s003.docx]

**S2 Table Specific primers used for amplification and sequencing of S7 sequences**

| Primer | Sequence (5’-3’) | Location |
| --- | --- | --- |
| S7-F-1 | CGACCTGTCTGGACCAGTACAT | 12-33 |
| S7-1-1 | AGCAGAACGATACATCGAAGCG | 1208-1229 |
| S7-2-1 | TGCTCCTACTGAGTTGCCTG | 1087-1106 |
| S7-R-1 | ACATCAGCTGCATTTCGACC | 2172-2191 |
