# Supplementary material for: Molecular Genetic Analysis and Evolution of Segment 7 in Rice Black-Streaked Dwarf Virus in China
Source: PLoS One. 2015 Jun 29;10(6):e0131410. doi: 10.1371/journal.pone.0131410 (PMC4488072; doi:10.1371/journal.pone.0131410)
Supplement: S3 Table — (DOCX) [file pone.0131410.s004.docx]

**S3 Table Relative synonymous codon usage (*RSCU*) values for each codon in S7-1 and S7-2**

| Amino acid | S7-1 | | | | | S7-2 | | | | |
| --- | --- | --- | --- | --- | --- | --- | --- | --- | --- | --- |
|  | Codon | RSCU-High | Bias-CU | RSCU-Low | Bias-CU | Codon | RSCU-High | Bias-CU | RSCU-Low | Bias-CU |
| Ala | GCA | 0.60 | 15 | 0.80 | 20 | GCA | 0.35 | 4 | 0.89 | 10 |
| Ala | GCC* | 1.00 | 25 | 0.40 | 10 | GCC | 0.00 | 0 | 0.09 | 1 |
| Ala | GCG | 0.20 | 5 | 0.20 | 5 | GCG@ | 0.52 | 6 | 0.00 | 0 |
| Ala | GCU | 2.20 | 55 | 2.60 | 65 | GCU | 3.13 | 36 | 3.02 | 34 |
| Arg | AGA | 3.00 | 50 | 2.53 | 40 | AGA | 2.14 | 25 | 1.27 | 15 |
| Arg | AGG | 0.30 | 5 | 0.32 | 5 | AGG | 0.86 | 10 | 2.11 | 25 |
| Arg | CGA | 0.90 | 15 | 1.20 | 19 | CGA | 1.20 | 14 | 1.27 | 15 |
| Arg | CGC | 0.00 | 0 | 0.82 | 13 | CGC | 0.43 | 5 | 0.42 | 5 |
| Arg | CGG | 0.00 | 0 | 0.06 | 1 | CGG@ | 0.51 | 6 | 0.00 | 0 |
| Arg | CGU@ | 1.80 | 30 | 1.07 | 17 | CGU | 0.86 | 10 | 0.93 | 11 |
| Asn | AAC | 0.52 | 30 | 0.44 | 24 | AAC | 0.48 | 24 | 0.48 | 25 |
| Asn | AAU | 1.48 | 85 | 1.56 | 86 | AAU | 1.52 | 76 | 1.52 | 80 |
| Asp | GAC | 0.32 | 20 | 0.38 | 24 | GAC | 0.54 | 35 | 0.62 | 40 |
| Asp | GAU | 1.68 | 105 | 1.62 | 101 | GAU | 1.46 | 95 | 1.38 | 90 |
| Cys | UGC | 0.75 | 15 | 0.75 | 15 | UGC | 0.00 | 0 | 0.10 | 1 |
| Cys | UGU | 1.25 | 25 | 1.25 | 25 | UGU | 2.00 | 20 | 1.90 | 20 |
| Gln | CAA | 1.63 | 49 | 1.80 | 54 | CAA | 1.67 | 25 | 1.67 | 25 |
| Gln | CAG | 0.37 | 11 | 0.20 | 6 | CAG | 0.33 | 5 | 0.33 | 5 |
| Glu | GAA | 1.60 | 64 | 1.75 | 70 | GAA | 1.57 | 74 | 1.47 | 70 |
| Glu | GAG | 0.40 | 16 | 0.25 | 10 | GAG | 0.43 | 20 | 0.53 | 25 |
| Gly | GGA | 1.07 | 20 | 1.07 | 20 | GGA | 3.23 | 21 | 3.20 | 20 |
| Gly | GGC | 0.43 | 8 | 1.07 | 20 | GGC | 0.00 | 0 | 0.00 | 0 |
| Gly | GGG | 0.05 | 1 | 0.00 | 0 | GGG | 0.00 | 0 | 0.00 | 0 |
| Gly | GGU | 2.45 | 46 | 1.87 | 35 | GGU | 0.77 | 5 | 0.80 | 5 |
| His | CAC | 0.86 | 15 | 1.11 | 20 | CAC | 0.29 | 5 | 0.34 | 5 |
| His | CAU | 1.14 | 20 | 0.89 | 16 | CAU | 1.71 | 30 | 1.66 | 24 |
| Ile | AUA | 0.53 | 20 | 0.52 | 20 | AUA@ | 0.95 | 35 | 0.55 | 20 |
| Ile | AUC | 0.66 | 25 | 0.86 | 33 | AUC | 0.71 | 26 | 0.52 | 19 |
| Ile | AUU | 1.82 | 69 | 1.62 | 62 | AUU | 1.34 | 49 | 1.94 | 71 |
| Leu | CUA | 0.50 | 15 | 0.50 | 15 | CUA* | 0.44 | 14 | 0.00 | 0 |
| Leu | CUC | 0.47 | 14 | 0.67 | 20 | CUC | 0.16 | 5 | 0.16 | 5 |
| Leu | CUG@ | 0.17 | 5 | 0.00 | 0 | CUG | 0.29 | 9 | 0.47 | 15 |
| Leu | CUU | 1.37 | 41 | 1.17 | 35 | CUU | 0.95 | 30 | 0.94 | 30 |
| Leu | UUA | 2.33 | 70 | 2.33 | 70 | UUA | 2.76 | 87 | 2.86 | 91 |
| Leu | UUG | 1.17 | 35 | 1.33 | 40 | UUG | 1.40 | 44 | 1.57 | 50 |
| Lys | AAA | 1.76 | 75 | 1.78 | 80 | AAA | 1.43 | 50 | 1.43 | 50 |
| Lys | AAG | 0.24 | 10 | 0.22 | 10 | AAG | 0.57 | 20 | 0.57 | 20 |
| Met | AUG | 1.00 | 40 | 1.00 | 40 | AUG | 1.00 | 45 | 1.00 | 45 |
| Phe | UUC | 0.65 | 26 | 0.63 | 25 | UUC | 0.37 | 21 | 0.54 | 31 |
| Phe | UUU | 1.35 | 54 | 1.38 | 55 | UUU | 1.63 | 94 | 1.46 | 84 |
| Pro | CCA | 1.00 | 20 | 1.14 | 21 | CCA | 0.00 | 0 | 0.00 | 0 |
| Pro | CCC | 0.45 | 9 | 1.08 | 20 | CCC | 0.63 | 9 | 1.41 | 19 |
| Pro | CCG | 0.55 | 11 | 0.22 | 4 | CCG | 0.14 | 2 | 0.00 | 0 |
| Pro | CCU | 2.00 | 40 | 1.57 | 29 | CCU | 3.23 | 46 | 2.59 | 35 |
| Ser | AGC | 0.53 | 17 | 0.51 | 17 | AGC | 0.95 | 20 | 0.51 | 11 |
| Ser | AGU | 2.01 | 64 | 2.04 | 68 | AGU | 1.86 | 39 | 2.26 | 49 |
| Ser | UCA | 1.10 | 35 | 1.20 | 40 | UCA | 0.38 | 8 | 0.65 | 14 |
| Ser | UCC | 0.47 | 15 | 0.45 | 15 | UCC | 0.00 | 0 | 0.28 | 6 |
| Ser | UCG@ | 0.16 | 5 | 0.00 | 0 | UCG* | 0.33 | 7 | 0.00 | 0 |
| Ser | UCU | 1.73 | 55 | 1.80 | 60 | UCU | 2.48 | 52 | 2.31 | 50 |
| Thr | ACA | 0.89 | 20 | 0.89 | 20 | ACA | 0.90 | 20 | 0.47 | 10 |
| Thr | ACC | 0.84 | 19 | 1.11 | 25 | ACC | 0.00 | 0 | 0.24 | 5 |
| Thr | ACG | 0.00 | 0 | 0.00 | 0 | ACG | 0.00 | 0 | 0.24 | 5 |
| Thr | ACU | 2.27 | 51 | 2.00 | 45 | ACU | 3.10 | 69 | 3.06 | 65 |
| Trp | UGG | 1.00 | 10 | 1.00 | 10 | UGG | 1.00 | 35 | 1.00 | 35 |
| Tyr | UAC | 0.40 | 20 | 0.70 | 35 | UAC | 0.76 | 19 | 0.70 | 19 |
| Tyr | UAU@ | 1.60 | 80 | 1.30 | 65 | UAU | 1.24 | 31 | 1.30 | 35 |
| Val | GUA | 0.73 | 20 | 1.09 | 30 | GUA | 0.63 | 17 | 0.57 | 15 |
| Val | GUC | 0.22 | 6 | 0.76 | 21 | GUC | 0.19 | 5 | 0.76 | 20 |
| Val | GUG | 0.36 | 10 | 0.18 | 5 | GUG | 0.48 | 13 | 0.95 | 25 |
| Val | GUU* | 2.69 | 74 | 1.96 | 54 | GUU* | 2.70 | 73 | 1.71 | 45 |
